# Feasibility, acceptability and appropriateness of a reproductive patient reported outcome measure for cancer survivors

**DOI:** 10.1371/journal.pone.0256497

**Published:** 2021-08-27

**Authors:** Brigitte Gerstl, Christina Signorelli, Claire E. Wakefield, Chantelle D’Souza, Rebecca Deans, Tejnei Vaishnav, Karen Johnston, Kristen A. Neville, Richard J. Cohn, Antoinette Anazodo

**Affiliations:** 1 Kids Cancer Centre, Sydney Children’s Hospital, Sydney, NSW, Australia; 2 Behavioural Sciences Unit, Kids Cancer Centre, Sydney Children’s Hospital, Sydney, NSW, Australia; 3 School of Women’s and Children’s Health, UNSW Medicine, University of New South Wales, Sydney, Australia; 4 Department of Gynaecology, the Royal Hospital for Women, Sydney, NSW, Australia; 5 Fertility Research Centre, the Royal Hospital for Women, Sydney, NSW, Australia; 6 Department of Endocrinology, Sydney Children’s Hospital, Sydney, NSW, Australia; 7 Nelune Comprehensive Cancer Centre, Prince of Wales Hospital, Sydney, NSW, Australia; University of Adelaide, AUSTRALIA

## Abstract

**Background:**

Cancer patients can experience a number of reproductive complications as a result of cancer treatment and may benefit from reproductive preventative health strategies. A Reproductive Survivorship Patient Reported Outcome Measure (RS-PROM) is not currently available but could assist patients address reproductive concerns.

**Purpose:**

To develop and test the acceptability, feasibility and appropriateness of a RS-PROM tool to be used to assess reproductive needs of cancer survivors aged 18–45 years.

**Methods:**

We reviewed the outcomes of a recently published audit of reproductive care provided in our cancer survivorship clinic to identify gaps in current service provided and used this along with available validated reproductive measures, to develop this pilot RS-PROM. Survivors aged 18–45 years either attending the SCH survivorship clinic over a 1-year period or participants on the Australasian Oncofertility Registry (AOFR) who had agreed to be contacted for future research studies were asked to complete the RS-PROM and a questionnaire on the acceptability, feasibility and appropriateness of content included.

**Results:**

One-hundred and fifty patients participated (61.3% females). Median age at cancer diagnosis was 24.5 years (range: 2–45 years). Eighty percent of participants reported the length of the RS-PROM was “just right”, 92% agreed they would not mind completing the RS-PROM and 92.7% were willing to answer all questions, with 97% agreeing that the RS-PROM would be an important tool in addressing difficult sexual/reproductive topics concerning with healthcare professionals.

**Conclusion:**

The large majority of survivors participating in our pilot study found the RS-PROM to be an acceptable, feasible and useful tool to assist discussions of their sexual and reproductive health concerns and experiences with their clinical team.

## Introduction

Improvements in treatment of cancer patients have led to significant improvements in survival rates [1,2]. One of the most devastating effects of cancer treatment include the late-effects consequences on a patient’s fertility, ability to have children in the survivorship period, their increased risks of medical complications (cardiovascular and bone health) and heightened psychological distress and anxiety (affecting relationships with a partner, sexuality and quality of life) [3,4]. A variety of concerns around their sexual and reproductive health in the survivorship period are reported in survivors [5,6] and their un-met need for opportunity and to address these [5].

The gonadotoxic effects of cancer treatment on a patient’s reproductive potential are variable. Risks will vary according to cancer type and stage, age at diagnosis, treatment received as well as a patient’s previous reproductive history and previous endocrine issues [7]. Survivors who received cancer therapies, more specifically chemotherapy and/or radiotherapy, may experience subfertility or infertility and may also be at higher risk for also experiencing adverse obstetric and birth outcomes including miscarriage, preterm birth and low birth weight babies [8–13]. Treatment can also cause temporary or permanent hormonal changes that may make it difficult for a survivor to have or enjoy sex [14].

Current models of survivorship care do not routinely offer reproductive health care beyond some discussions around fertility [3,15–19]. Many survivors report that they do not receive adequate information or are not given the opportunity to discuss the potential effects of their cancer treatment on their fertility as well as their concerns about loss of sexual intimacy following cessation of treatment and future parenthood [20,21]. Other late-effects consequences of cancer treatment can include heightened anxiety about meeting a new intimate partner, poor self-esteem surrounding changes in body image, lack of confidence and side effects associated with medication that can negatively affect a relationship [14].

### Description of PROMs

Patient Reported Outcome Measures (PROMs) are instruments (questionnaires) that capture a patient’s experience regarding clinical outcomes associated with their diagnosis, treatment and care [22]. Reproductive health, sexual and psychosexual health are topics that many patients and cancer clinicians find difficult to raise and address. A streamlined patient reported assessment tool may be able to provide patients with an opportunity to self-assess their disease symptoms, issues associated with treatment and their outcomes in the survivorship period [23]. Additionally, PROMs can enable clinicians to understand the unique experience of a patient in order to support personalised care [24].

The development of a reproductive survivorship PROM (RS-PROM) may allow patients an opportunity to discuss reproductive symptoms or concerns to improve a consultation with a health care professional (HCP), which could enhance patient care. To date there have been no PROMs developed to evaluate cancer patients’ perspectives in relation to their current or future sexual and reproductive concerns.

### Aims

The aims of this study were:

- To develop an RS-PROM to help identify the sexual and reproductive concerns experienced by cancer patients in the survivorship period.
- To assess the feasibility, appropriateness, acceptability, concerns and usefulness of questions included in the RS-PROM.
- To evaluate the potential usefulness for integration of an RS-PROM into a cancer survivorship clinic setting.

## Material and methods

This research follows on from an initial survivorship study which explored the reproductive care of childhood and adolescent cancer survivors, aged from 0–18 years at diagnosis who attended a survivorship clinic over a 12-year period [25]. From this study, we were able to identify a number of themes relating to sexual and reproductive care provided from both a patient and clinician perspective [25].

### Development of the PROM

We recognised that the reproductive needs of cancer patients might be different based on age, gender and sexuality so for the first reproductive PROM development we decided to develop and pilot the RS-PROM on a cohort of adult survivors diagnosed with a cancer during childhood, adolescent and young adult (AYA) and adulthood who were aged 18–45 years at the time of completing the questionnaire. Having audited our survivorship clinic service in phase 1, we again reviewed the literature for available validated measures, which assessed the key areas of reproductive needs which were identified from the survivorship clinic.

The developed PROM was uploaded to the Qualtrics [26] on-line survey platform by two researchers (CD’S and BG). The PROM was divided into sections based on the measures that were included and heading descriptions that provided information and instructions to participants. The RS-PROM was reviewed by members of our research team and tested by a multidisciplinary team of health care professionals and consumers who tested the flow of questions and time taken to complete the RS-PROM prior to the commencement of the formal pilot. Following discussions, the RS-PROM was updated and divided into two separate PROMs for male and female participants.

### Evaluation of the RS-PROM

Patients were asked to complete the RS-PROM in relation to their own experience and then complete a questionnaire on the feasibility (ease of completion), appropriateness (clear and understandable), acceptability (layout of PROM and time taken), concerns and usefulness of completing the RS-PROM (feelings on the content and use of the PROM). The questionnaire included an opportunity for patients to provide additional free-text comments about their experience using the RS-PROM. Illustrative quotes from participants’ free-text responses are included in-text where appropriate.

### Instruments

The RS-PROM utilised existing validated measures to address medical and psychological reproductive health concerns that affect cancer survivors which were identified following a systematic review of the literature (Appendix A) [5,6,27,28]. The research team also developed a series of questions to evaluate puberty, hormonal function, contraception and questions which assessed fertility and future pregnancies.

The RS-PROM comprised basic demographic and clinical information. Sociodemographic information included: patient name, date of birth, gender and sexuality. Clinical features included age at cancer diagnosis, cancer type, and cancer treatment received. The Sexuality Scale (also referred to as the ‘Kinsey scale’ [29]) was used to describe a person’s sexual orientation based on the respondent’s response at a given time. The categorical version of this scale has four options: heterosexual, bisexual, gay/lesbian, other. The research team modified the scale to include an additional option “I prefer not to answer.”

The Body Image Scale (BIS) [30] provided an opportunity to better understand a patient’s reproductive concerns following cancer treatment. The BIS comprised of 10 items and the items were scored on a five-point Likert scale with a range from one to five. Lower scores indicating poor body image.

The 22-item EORTC Sexual Health Questionnaire (EORTC-SHQ-C22) was used to assess the sexual function of a cancer patient [31]. The multidimensional quality of life instrument included both sexual functioning and psychosexual questions, with items on sexual satisfaction, sexual pain, and single items representing an integrative approach [32]. Higher scores in the functioning scales indicated better functioning, whereas higher scores in the symptom scales indicated severity with each item.

The Reproductive Concerns After Cancer (RCAC) Scale [33] included 18 items, and consisted of six subscales each with three items included. This tool measured fertility potential, partner disclosure of fertility status, child’s health, personal health, acceptance of possible infertility and becoming pregnant. Items were scored on a five-point Likert scale with a range from one to five. Lower scores indicating a stronger agreement with an item.

Other components of the RS-PROM included a section on pubertal development which comprised of ten questions centred on the impact of a cancer diagnosis or treatment on a survivor’s pubertal development. The Emotion Thermometer tool was also incorporated to assess responder levels for depression, anxiety, distress, anger and need for help via a visual ‘thermometer’ scale with a range from zero to ten, where higher scores indicating a greater impact associated with a feeling [34,35].

### Assessment of the RS-PROM

#### Patient feasibility to complete the RS-PROM prior to a clinic consultation

Participants were asked to comment on the types of questions that were included in the RS-PROM and to provide free-text feedback around completion of the RS-PROM prior to a scheduled clinic appointment. Participants were also asked to comment as to whether the RS-PROM would be helpful to stimulate discussions about topics related to their sexual and reproductive concerns with a health professional.

#### Acceptability (layout of the RS-PROM and time taken)

Participants were asked to comment about the acceptability of time taken to complete the RS-PROM (less than 5 minutes, 5–10 minutes, 10–15 minutes, 15–20 minutes, 20–25 minute, 25–30 minutes and greater than 30 minutes). Participants were also offered the opportunity to provide free-text comments about the acceptability of the length and the structure of the RS-PROM.

### Appropriateness

Participants were requested to provide free-text comments and suggestions about the appropriateness of the wording of questions and instructions, included in the RS-PROM.

#### Concerns and usefulness of content (feelings on the content and use of the RS-PROM)

Participants were asked to provide comments about the relevance and appropriateness of questions and content included under each theme presented in the RS-PROM which was relative to their cancer reproductive survivorship experiences. Participants were also asked to comment about whether their personal health documentation (medical records, Health Summary/Passport or other long term follow-up documents) were required to answer certain questions to assist with completion of the RS-PROM. They were also asked as to whether assistance was required by an external source to support the completion of the RS-PROM process (by myself, friend, and partner).

### Inclusion criteria

Inclusion criteria included: (i) cancer survivors who were currently aged >18 and ≤45 years and had completed active cancer treatment and diagnosed more than 5 years ago, (ii) had attended a survivorship clinic at the Sydney Children’s Hospital Survivorship Clinic in Sydney Australia for five years, (iii) were registered with the Australasian Oncofertility Registry (AOFR) [36] and had agreed to be contacted for future research studies in relation to oncofertility. The Sydney Children’s Hospital survivorship clinic follows children and adults that were previously treated at the Sydney Children’s Hospital for a childhood or adolescent cancer aged 0–18 years of age.

### Exclusion criteria

Patients who did not meet the above inclusion criteria or where a clinician did not feel the patient would be appropriate to be contacted in relation to this study were excluded from participating in the study.

### Recruitment

The patient’s medical clinician, nurse navigator or study coordinator approached patients at their routine consult at the survivorship clinic and invited them to participate. Survivors registered with the AOFR were contacted by phone to offer the survey and via mail. Patients were followed-up by phone, following a mail out of the survey to determine if they were interested in participating and to set-up a time for a phone interview. Overall, there were 214 survivors approached to participate and 150 agreed to participate, a breakdown of patients recruited via the survivorship clinic compared to the AOFR was not collected. If the patient decided to participate, by signing the consent form, they were offered an opportunity to complete the tool (RS-PROM) either via an on-line link to a gender-specific questionnaire or using a hard-copy of the tool. Creating a secure link to the tool on-line allowed participants to complete the tool in different settings.

### Statistical analysis

Data were downloaded from Qualtrics and transferred into a Microsoft Excel spreadsheet and other databases. Descriptive statistics, including frequencies and proportions for categorical variables and mean, median and ranges for continuous variables were reported. Groups were compared using the Chi-squared (*x*^2^) test (or Fisher’s exact test) for categorical variables. Analyses were carried out in STATA 14.2 (StataCorp, College Station, TX, USA) [37]. Qualitative data were recorded on an excel spreadsheet and grouped by theme.

### Ethics approval

Ethics approval for this study was obtained from the Sydney Children’s Hospitals Network Human Research Ethics Committee, HREC reference LNR/16/SCHN/396.

## Results

### Demographic information

There were 150/214 (70.1%) cancer survivors who consented to participate. Participants included 58 (38.7%) males and 92 (61.3%) females. Participants’ median age at cancer diagnosis was 24.5 years (range: 2–45 years). The median time from a cancer patient finishing treatment to completion of the RS-PROM was 23 years [range: 2–45 years]). The most frequent cancer diagnoses reported by participants were breast cancer (n = 29, 19.3%) lymphoma (n = 28, 18.7%) leukaemia (n = 22, 14.7%), testicular cancer (n = 19, 12.7%) and bone or soft tissue cancers (n = 17, 11.3%).

### RS-PROM evaluation

**Fig 1:**
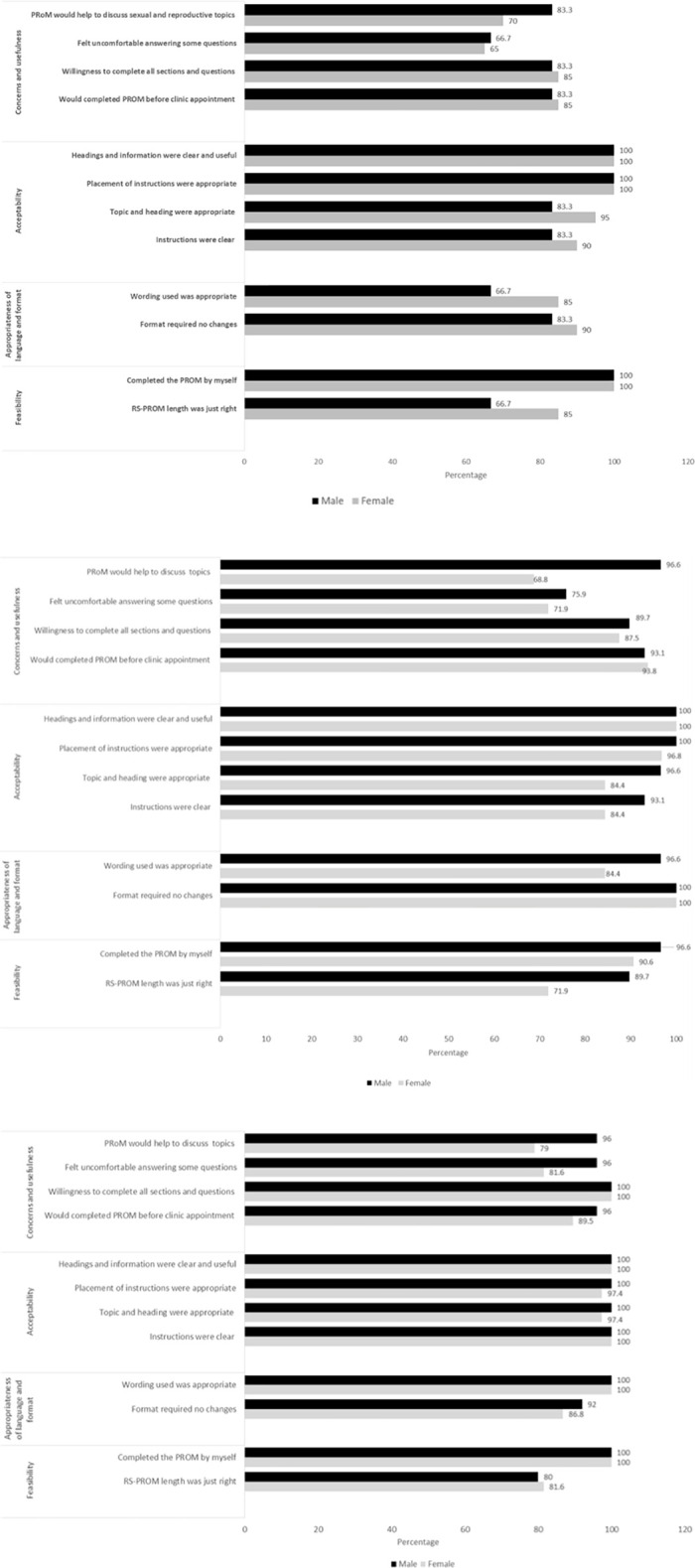
a. Paediatric childhood cancer survivor’s responses to themes in the RS-PROM. b. AYA cancer survivor’s responses to themes in the RS-PROM. c. Adult cancer survivor’s responses to themes in the RS-PROM.

The following four themes were evaluated as part of the RS-PROM: (i) feasibility (ease of completion), (ii) appropriateness (clear and understandable), (iii) acceptability (layout of PROM and time taken) and (iv) concerns and usefulness of completing the RS-PROM (feelings on the content and use of the PROM). Outcomes from the most commonly reported responses to each theme are presented graphically in Fig 1A–1C.

#### Feasibility for the patient to complete

Participants most commonly took between 10–15 minutes (28.7%) (males: 36.7% *versus* females: 23.3%) to complete the RS-PROM followed by 15–20 minutes (22%) (males: 38.3% *versus* females: 11.1%) and 25–30 minutes (16.7%) (males: 3.3% *versus* females: 25.6%) (p<0.001). When asked to comment on the length of the PROM, most participants (80.7%) felt that the length of the questionnaire was “just right” (males: 85.7% *versus* females: 77.7%, p = 0.34). However, 27 (18%) participants (males: 14.3% *versus* females: 20.2%, p = 0.34) felt that the length of the questionnaire was “too long”. When participants were asked whether they reviewed their own personal medical documents (medical records, Health Summary/Passport or other long-term follow-up documents) to complete the questions within the RS-PROM, 5.3% stated that personal medical documentation were required to aid in ascertaining specific information to complete some questions. Participants most commonly stated that they completed the RS-PROM themselves (97.3%) (males: 100% *versus* females: 91.1%, p = 0.02). One female participant reported requiring assistance from a partner and two female participants received assistance from a family member (1.3%).

#### Appropriateness of language and format of RS-PROM

There was also a strong agreement (92.7%) from participants that the wording used in the questionnaire was clear, understandable and appropriate to their cancer and reproductive survivorship experience. Amongst participants who felt the wording of the RS-PROM was appropriate, 91.1% (n = 82) were female and 95.0% (n = 57) were male (p = 0.37). One participant suggested “perhaps define the term ‘sexually active’, I left a relationship before my diagnosis and haven’t been in one since” (male, aged 27 years, testicular cancer) and another stated that “the only negative experience I had was during fertility preservation, so you could include questions tailored towards that; everything else included was appropriate” (female, aged 23 years, non-Hodgkin’s lymphoma).

Less than 10% of participants indicated that changes to the format of the RS-PROM were required (males: 5.0% *versus* females: 7.8%, p = 0.50). Helpful feedback reported by participants included “page breaks when completing tables can make referring back to table heading options tricky” (female, aged 42 years, breast cancer); “you need to add question numbers next to each question” (female, aged 33 years, Hodgkin’s lymphoma) and “add more free text boxes in order to be able to elaborate on one’s individual circumstances” (female, aged 31 years, breast cancer).

Almost all participants were in agreement that there were no difficulties understanding the words or sentences within the RS-PROM (94.7%), with only 5.3% of participants reporting difficulties understanding the words or sentence structure in some of the questions (males: 8.3% *versus* females: 3.3%, p = 0.18).

#### Acceptability

Participants most commonly stated that the RS-PROM provided adequate and clearly defined instructions for completion of the RS-PROM (93.3%) (males: 95.0% *versus* females: 92.2%, p = 0.50). A strong agreement (92.7%) was also reported for the number of response categories, to select from for each question, was adequate (92.7%) (males: 96.7% *versus* females: 90.0%, p = 0.13). One participant expressed that “some of the questions in the tables cannot always be accurately answered by the options provided” (female, aged, 32 years, breast cancer).

Overall, participants (94%) agreed that the topics and headings included in the RS-PROM were appropriate (males: 96.7% *versus* females: 92.2%, p = 0.26). Almost all (98.7%) were in agreement that the placement of instructions in relation to each question was appropriate (males: 100% *versus* females: 97.8%, p = 0.25). One patient stated “Yes, the language used is easy to understand; no medical jargon. I liked how you defined fertility and made it clear that there were no ‘right’ or ‘wrong’ answers” (female, aged, 29 years, breast cancer).

All participants (100%) agreed that the headings and information at the start of each section were clearly displayed and useful. Feedback in relation to the headings of each section included “Yes, made sure you had an idea of the topics/questions that were going to be addressed” (female, 25 years, Hodgkin’s lymphoma) and “Yes all headings were articulately presented and made sections easy to follow throughout” (female, aged 33 years, breast cancer).

#### Concerns and usefulness

Participants (92%) expressed that that they “would not mind” completing the RS-PROM prior to attending their scheduled clinic appointment (males: 95.0% *versus* females: 95.0%, p = 0.27). One participant reported that “If I had a clinic appointment, I would think it was a good way to collate important information to appropriately target discussions” (female, aged 33 years, Hodgkins lymphoma); while another wrote “I would definitely prefer to complete the PROM before any clinic appointments/cancer treatments as during that time things are already overwhelming” (female, aged 19 years, ovarian tumour).

Participants stated that they were willing to complete all of the questions in each section of the RS-PROM (92.7%) (males: 93.3% *versus* females: 92.2%, p = 0.80). One participant who felt unwilling to answer all of the questions in the PROM stated “maybe the orgasm questions in the ‘Sexual Health Function’ section were a bit embarrassing, not something that my doctor might need to know” (female, aged 32 years, breast cancer). Another participant reported that they were “fine” to answer the questions included in the PROM, but recommended considering the suitability of the setting in which the RS-PROM was offered, suggesting that clinicians be mindful of other people who were also in attendance at a consultation when offering the RS-PROM. The participant commented that, “I would feel ok with it. If my partner or family were with me, I would feel uncomfortable answering the questions on sexual satisfaction/libido etc. and body image truthfully for fear of embarrassment, worrying them or hurting their feelings. I also felt a little emotional when reflecting on my body image/scars and ideally would not want to answer these questions at home” (female, aged 29 years, breast cancer).

When asked whether there were any questions that made participants feel uncomfortable, overall 22% of participants felt uncomfortable with some of the questions; with more female participants feeling uncomfortable about answering some of the specific sexual health and reproductive questions, about ‘sexual activity’ and ‘pain during sex,’ compared with males (males: 16.7% *versus* females: 25.6%, p = 0.19). One participant commented “maybe place greater emphasis on confidentiality, it might have helped me feel less awkward answering the more personal sex and body questions” (female, aged 32 years, breast cancer).

Overall 82% of participants felt that the RS-PROM would help to bring up sexual/reproductive topics, that they would not normally feel comfortable discussing with their treating doctor; with significantly more males responding favorably to this question compared with female participants (males: 95.0% *versus* females: 73.3%, p<0.001). Comments included that “the PROM might induce more questions about fertility;” “won’t be as awkward bringing up the topic or questions during the consultation”; “I think it could be a good conversation starter with my doctor or the nurses”; “It will make me more open to talking about my reproductive health with my medical team;” “I currently seek support regarding my reproductive health and have for some time, so these questions were timely” and “having doctors and specialists know about my previous history and areas that I am concerned about, makes me more relaxed about having discussions of this nature.”

## Discussion

To our knowledge, this is the first RS-PROM developed to identify the reproductive symptoms or concerns that may affect a cancer patient in the survivorship period. It was well accepted by participants and found almost universally to be a desirable tool to assist in oncofertility care. Feedback from users was constructive and will guide modifications of future tools to be used in survivorship clinics. Considering the difficulty many patients find in raising these concerns with their health care providers in current clinical settings we feel this tool may fulfil an important role in addressing survivors oncofertility needs in routine survivorship care.

The RS-PROM is novel as it may facilitate the provision of value-based care in which health services and procedures can be motivated by patient preferences, needs, and health outcomes [38]. It allows the patient to address and discuss topics around their unique health experiences. Using a PROM can also support the construct that patients want to be more actively involved in the decision-making process that affects their overall health and health related quality of life.

Our study reports that the majority of participants were in agreement (96.7%) that the RS-PROM would heighten patient-clinician engagement and improve discussions with their health professional team. They strongly agreed (92.7%) that the wording and language displayed in the RS-PROM was clear, appropriate and acceptable and felt that implementing the RS-PROM into a clinical setting would be beneficial.

Nearly full agreement was received by participants (92.7%) in relation to the appropriateness of the content of the RS-PROM. Participants felt that the RS-PROM addressed specific aspects of the cancer experience and indicated that they would not mind completing the RS-PROM prior to a scheduled clinic appointment. Participants (93.3%) also indicated that the instructions integrated throughout were clear and helpful. Almost all participants felt that the placement of the instructions was adequate and aided in the ease of completion of the RS-PROM. Additionally, including headings in tables where the questions went over the page as well as numbering each of the questions were general administrative comments that the research team has taken into consideration to improve the RS-PROM.

The use of PROMS within clinical practice often requires the use of electronic medical systems to facilitate the ease of collection and analysis of data [23]. A PROM may be useful for providing direct feedback to the clinician, prior to a scheduled appointment to facilitate patient focused discussions during the consultation. Most participants (92%) indicated that they felt “fine” to complete the RS-PROM before attending a clinic appointment with their treating specialist team. The RS-PROM may provide a useful avenue to help guide patient-clinician conversations around a patient’s sexual and reproductive health concerns. Some participants expressed that those administering a RS-PROM should be mindful of having support personnel in attendance with a patient at appointments when offering the RS-PROM, particularly as sensitive topics such as ‘sexuality,’ ‘sex,’ ‘sexual function’ and ‘body image’ are addressed. Furthermore, patient concerns need to be considered when addressing and exploring themes that might be viewed as culturally or ethnically sensitive for some patients to discuss, particularly in light of the fact that some parents and family members attend consultations with a patient.

This RS-PROM can be utilised alongside existing methods of patient-centred decision making such as history taking, clinical examination and investigations to improve the patient-clinician interaction. In further studies the implementation of the RS-PROM as part of the clinic model of care will be studied to not only test the benefits to patients completing it prior to the consultation but also the success of the implementation procedure.

## Strengths and limitations

### Strengths

One of the key strengths of this study includes the development and pilot testing of the first RS-PROM which explores, identifies and highlights the reproductive needs of adult survivors in relation to their cancer experience. Incorporation of the patient perspective to help guide and develop the RS-PROM was instrumental to its development and may have contributed to the successful response we received from patients’.

### Limitations

The RS-PROM was only piloted in adult survivors, and more work would be needed for use and applicability in younger cancer patient cohorts. Additionally, we did not collect data on the age of the survivors at the time of completing the RS-PROM, although all survivors were over eighteen years of age at the time of completing the RS-PROM. Hence, this will be included in an updated RS-PROM.

Furthermore, the RS-PROM was piloted in English and so further research would be required to better understand the views of a cultural and linguistically diverse cohort of cancer patients. Moreover, this study is limited in its ability to implement the RS-PROM in routine clinical practice and so future research studies are intended to explore this further.

## Future directions

The next step would be to study the implementation of the RS-PROM prior to a scheduled survivorship clinic consultation and then test the difference in patient and clinician satisfaction about the consultation as well as studying the process of implementation.

## Conclusion

The RS-PROM provides a comprehensive evaluation of a cancer survivor’s sexual and reproductive health post-cancer treatment, addressing multiple themes in relation to reproductive care, and was well accepted and liked by participants. The incorporation of patient feedback integrated into this RS-PROM, highlights areas that require further assessment and improvement in order to tailor a measurement tool that can address a patient’s individual experience. Findings that have emerged from this study may better enhance patient and clinician interactions around sensitive topics, which have wider implications for guiding health care policy.

## Supporting information

S1 Fig Female PROM survey. (PDF)

S2 Fig Male PROM survey. (PDF)

S1 Data PLOS ONE data repository. (XLSX)
