# Supplementary material for: Feasibility, acceptability and appropriateness of a reproductive patient reported outcome measure for cancer survivors
Source: PLoS One. 2021 Aug 27;16(8):e0256497. doi: 10.1371/journal.pone.0256497 (PMC8396734; doi:10.1371/journal.pone.0256497)
Supplement: S2 Fig — (PDF) [file pone.0256497.s002.pdf]

Introduction to survey

Survivorship PROM (MALES)

Thank you for consenting to complete the 'Reproductive Patient Reported Outcome Measure' (PROM).

We are aiming to develop this measure to help patients identify any reproductive symptoms or concerns they may have. This will allow us to identify which additional patients would benefit from being seen in this clinic.

We know patients can find it very embarrassing to discuss issues related to reproduction especially when they feel that something is not quite right. We also know that health care professionals can assume everything is okay unless patients bring up their symptoms or concerns. This PROM will help the medical team address patient's concerns.

The PROM has questions that cover different areas of reproduction (puberty, hormonal function, sexual health, contraception, fertility and future parenthood).

Not all the questions will relate to your personal situation and so you will have the opportunity to select no or not applicable (N/A) and move to the next question.

In the final version, the PROM will be completed prior to clinic and a copy will be given to the doctor so they can have a conversation based on what the patient's concerns are.

In this version we are only testing the suitability of the questions, how easy it is to understand the questions and complete the questionnaire. As with all your consultations, all information collected as part of this PROM is strictly confidential and will not be shared with your partner or family member/s unless you choose to complete the questionnaire with them. We estimate it will take approximately 15 minutes to complete.

After reviewing the PROM we would be grateful if you could spend ten minutes answering

questions about the PROM. We want this to benefit your future visits so please provide any additional comments you think will be useful.

I am 18 years or older

Yes

No

I consent to participating in this PROM

Yes

No - you will be directed to exit this survey

Demographic information

Name

Date of birth

Diagnosis

Age at diagnosis (years)

Email address

Relationship Status

In a relationship

Not in a relationship

Other

Sexuality

Sexuality

It is important for us to know your sexuality so that your clinician can tailor discussions surrounding your reproductive health. This information is strictly confidential and will not be shared with any partners or family members.

How would you describe your sexual orientation?

Heterosexual

Bisexual

Bisexual

Gay/Lesbian

Other

I prefer not to answer

Please specify other

Body Image

Body Image

This section helps us to understand how your body image may impact your reproductive concerns.

Please respond to all of the statements below by indicating, with a tick in the box, the response which best applies to you. There are no 'right' or 'wrong' answers.

During the last 4 weeks

	Not at all	A little	Quite a lot	Very much
Have you been feeling self-conscious about your appearance?	☐	☐	☐	☐
Have you felt less physically attractive as a result of your disease or treatment?	☐	☐	☐	☐
Have you been dissatisfied with your appearance when dressed?	☐	☐	☐	☐
Have you been feeling less masculine as a result of your disease or treatment?	☐	☐	☐	☐
Do you find it difficult to look at yourself naked?	☐	☐	☐	☐
Have you been feeling less sexually attractive as a result of your disease or treatment?	☐	☐	☐	☐
Have you avoided people because of the way you felt about your appearance?	☐	☐	☐	☐
Have you been feeling the treatment has left your body less whole?	☐	☐	☐	☐
Have you felt dissatisfied with your body?	☐	☐	☐	☐

	Not at all	A little	Quite a lot	Very much
Have you been dissatisfied with the appearance of a scar/s from cancer treatment?	▢	▢	▢	▢

Puberty

Puberty

This section is about how your cancer diagnosis or treatment may have impacted your pubertal development.

Did you receive cancer treatment prior to entering into puberty?

Yes

No

Are you concerned that your sexual characteristics have not developed e.g. pubic hair, hair under armpits, your genetalia?

Yes

No

Did you need medication to go into puberty?

Yes

No

Hormone Treatment and Bone Health

Hormone Treatment and Bone Health

This section is about your hormone levels following your cancer diagnosis and treatment. We also ask questions about your bone health which may be related to low hormone levels.

Have you been investigated for hormonal problems, prior to coming to this clinic?

Yes

No

Do you take any replacement hormones?

Yes

No

What hormonal treatment do you take?

Who manages your hormone treatment?

Have you ever had a bone fracture following your cancer treatment?

Yes

No

Have you had a diagnosis of osteoporosis, brittle, weak or fragile bones?

Yes

No

Have you ever had a DEXA or bone density scan to look at your bone health?

Yes

No

Sexual Health and Function

Sexual Health and Function

In this section, we are evaluating your sexual function which will help guide the discussion held with your clinician. If you are not sexually active some of the questions may still be relevant to you so please read through all of the questions.

Please respond to all of the statements below by indicating the option that best applies to you. There are no 'right' or 'wrong' answers. Some questions may not be relevant to you, if you feel that these questions are not applicable, please select the N/A option.

Have you ever been sexually active?

Yes

No

Are you currently sexually active?

Yes

No

I prefer not to answer

During the last 4 weeks

	Not applicable	Not at all	A little	Quite a lot	Very much
How important to you is an active sex life?	☐	☐	☐	☐	☐
Have you had decreased libido (sexual desire)?	☐	☐	☐	☐	☐
Has sexual activity been enjoyable for you?	☐	☐	☐	☐	☐
Have you been satisfied with your ability to reach an orgasm?	☐	☐	☐	☐	☐

	Not applicable	Not at all	A little	Quite a lot	Very much
Have you been worried about being incontinent (urine/stool)?	☐	☐	☐	☐	☐
Has fatigue or a lack of energy affected your sex life?	☐	☐	☐	☐	☐
Has your cancer treatment affected your sexual activity?	☐	☐	☐	☐	☐
Have you felt pain during/after your sexual activity?	☐	☐	☐	☐	☐
Have you been worried that sex would be painful?	☐	☐	☐	☐	☐
Have you had communication with health professionals about sexual issues?	☐	☐	☐	☐	☐
Have you been satisfied with the communication about sexual issues between yourself and your partner(s)?	☐	☐	☐	☐	☐
Have you been worried that your partner(s) may cause you pain during sexual intercourse?	☐	☐	☐	☐	☐
Have you been satisfied with your level of intimacy?	☐	☐	☐	☐	☐
Have you felt insecure regarding your ability to satisfy your partner(s)?	☐	☐	☐	☐	☐
Have you been sexually active following your cancer diagnosis?	☐	☐	☐	☐	☐

	Not applicable	Not at all	A little	Quite a lot	Very much
To what extent did you experience sexual enjoyment?	☐	☐	☐	☐	☐
Have you been satisfied with your sex life?	☐	☐	☐	☐	☐

During the last 4 weeks, if applicable

	Not applicable	Not at all	A little	Quite a lot	Very much
Were you confident about obtaining and maintain an erection when you had sex?	☐	☐	☐	☐	☐
Were you concerned about having a shortened penis?	☐	☐	☐	☐	☐
Have you felt less masculine as a result of your disease or treatment?	☐	☐	☐	☐	☐
Have you been satisfied with your ability to reach an orgasm?	☐	☐	☐	☐	☐

Do you have any questions or concerns about sexual functioning that you would want your doctor to know about?

Would you like to receive any information about sexual health or dysfunction?

Yes

No

Contraception

Contraception

In this section we are interested in your current contraception use and history.

Are you or your partner currently using any contraception?

Yes

No

I prefer not to answer

What contraception are you or your partner using?

Would you like to receive any information about contraception?

Yes

No

Fertility and Future Pregnancies

Fertility and Future Pregnancies

This section is related to your fertility and desire to have a child in the future.

Fertility describes the ability of a couple, not using contraception, to become pregnant through sexual activity. Pregnancy is the act of carrying an embryo or fetus within the female body.

Did you have fertility preservation?
(Fertility preservation is the use of medical and surgical treatments to reduce the negative consequences of cancer treatment on a patient's fertility. This includes: sperm banking)

Yes

No

Are you and your partner currently pregnant or trying to get pregnant?

Yes

No

Was there ever a period in your life when you and your partner tried for one year or more to become pregnant without success?

Yes

No

Have you ever had medical tests to see whether or not you might have trouble having children?

Yes

No

Has a doctor ever found a reason/s why you or a partner was unable to get pregnant?

Yes

No

Did you ever take medication to help you or a partner try to get pregnant?

Yes

No

Have you and a partner ever become pregnant?

Yes

No

Would you like to receive information about fertility preservation or pregnancy or assisted reproductive technology?
(Assisted Reproductive Technology refers to technology and methods used to assist individuals to achieve a pregnancy. This includes: artificial insemination, donor

conception and in-vitro fertilisation.)

Yes

No

Fertility Concerns Following Cancer

Fertility Concerns Following Cancer

This section explores any concerns you have about your current or future fertility and how this impacts on your relationship and decisions to have a child(ren).

Please respond to all of the statements below by indicating how much you agree or disagree with each item, if applicable. There are no correct or incorrect answers.

	Strongly agree	Agree	Neither agree nor disagree	Disagree	Strongly disagree
I am afraid I won't be able to have any (more) children	☐	☐	☐	☐	☐
I am worried about my ability to get pregnant (again)	☐	☐	☐	☐	☐
I am concerned that I may not be able to have (more) children	☐	☐	☐	☐	☐
I worry about telling my (potential) spouse/partner that I may be unable to have children	☐	☐	☐	☐	☐
I am concerned that my (potential) spouse/partner will be disappointment if I can't get pregnant	☐	☐	☐	☐	☐

	Strongly agree	Agree	Neither agree nor disagree	Disagree	Strongly disagree
The thought of telling my (potential) spouse/partner that I may be unable to have children makes me uncomfortable	☐	☐	☐	☐	☐
I am worried about passing on a genetic risk for cancer to my children	☐	☐	☐	☐	☐
I am worried about how my family history might affect my children's health	☐	☐	☐	☐	☐
I am afraid my children would have a high chance of getting cancer	☐	☐	☐	☐	☐
I am scared of not being around to take care of my children someday	☐	☐	☐	☐	☐
Having (more) children will make me more nervous about getting cancer again	☐	☐	☐	☐	☐
I am cautious about having (more) children because I might not be around to raise them	☐	☐	☐	☐	☐
I can accept it if I'm unable to have (more) children	☐	☐	☐	☐	☐
I will be happy with life whether or not I have (more) children someday	☐	☐	☐	☐	☐
I will feel content if I do not have (more) children	☐	☐	☐	☐	☐

	Strongly agree	Agree	Neither agree nor disagree	Disagree	Strongly disagree
I am overwhelmed by the thought of trying to get pregnant (again)	☐	☐	☐	☐	☐
I worry that getting pregnant (again) would take too much time and effort	☐	☐	☐	☐	☐
It is stressful to think about trying to get pregnant (again)	☐	☐	☐	☐	☐

Emotion Thermometers

Emotion Thermometers

These visual scales are used to assess your experience of distress, anger, depression and anxiety in regards to your reproductive health as a result of your cancer diagnosis or treatment.

In the first four scales, please circle the number (0-10) that best describes how much emotional distress you have been experiencing in relation to reproductive health concerns over the past four weeks.
0= no distress and 10= extreme or high distress.

	0	1	2	3	4	5	6	7	8	9	10
Distress											
Anxiety											
Depression											
Anger											

Please indicate how much you need help for these concerns.

0= no distress and 10= extreme or high distress.

	0	1	2	3	4	5	6	7	8	9	10
Please slide across to indicate how much you need help											

Are you currently seeking help with these problems?

Yes

No

Do you require further help for these problems?

Yes

No

PROM Evaluation Questions

PROM Evaluation Questions

Thank you for reading through the Reproductive Patient Reported Outcome Measure (PROM). Thinking about your experience in answering this questionnaire, please answer the following questions. Your answers will help us make changes to improve the PROM. There are no correct answers, the research team are interested in hearing your views.

Is the wording used in questions and responses clear and appropriate?

Yes

No

Do you have any suggestions to improve the wording of questions/responses?

Were the questions appropriate to your cancer reproductive survivorship experiences?

Yes

No

Do you have any suggestions to improve the appropriateness?

Do the responses offer a clear distinction between choices?

Yes

No

Do you have any suggestions to improve the distinction between response choices?

Are the instructions for completing the questionnaire and selecting response options adequate?

Yes

No

Do you have any suggestions to improve the instructions?

Are the number of response options justified?

Yes

No

Do you have any suggestions to improve the number of response options?

Are the topic headings and questions appropriately ordered?

Yes

No

Do you have any suggestions to improve the order of topics?

Is the placement of instructions within the PROM adequate?

Do you think the information at the start of each heading was clear and useful?

Do you require new instructions for each question?

Would you make any changes to the format?

How long did it take to complete the questionnaire?

Less than 5 minutes

5-10 minutes

10-15 minutes

15-20 minutes

20-25 minutes

25-30 minutes

Greater than 30 minutes

Was the length of the questionnaire appropriate?

Too long

Just right

Too short

Was the format of the questionnaire adequate?

Yes

No

Do you have any suggestions to improve the format?

Did you have difficulty understanding the words or sentence structure?

Yes

No

Which words or sentence structures did you have difficulty understanding?

Did you need to consult your own records to complete the questionnaire?

Did you complete the questionnaire by yourself or with help from a parent/friend/partner?

How would you feel about completing this questionnaire prior to your clinic appointment?

Were there any questions that you were unwilling to answer?

What were the topic areas and do you have any suggestions to improve these questions?

Were there any questions that made you feel uncomfortable?

Yes

No

What were these topic areas and do you have any suggestions to improve these questions?

How do you think the PROM will make a difference to your reproductive consultation, if at all?

Would this PROM help to bring up topics that you would not feel comfortable discussing with your doctor?

Yes

No

Did the PROM help you identify your reproductive concern, if any?

Yes

No

No current concerns

How could we improve the PROM so it identifies these reproductive concerns, if any?

Did you have any additional feedback?

Powered by Qualtrics
